# Pulmonary vein isolation durability in a randomized trial comparing fluoroscopy-only vs. electroanatomic mapping strategies for pulmonary vein isolation with balloon-in-basket pulsed-field ablation

**DOI:** 10.1093/europace/euag244

**Published:** 2026-09-03

**Authors:** Daniel Rodríguez Muñoz, Isabel López-Alacid, Lucía Cobarro Gálvez, Ez Alddin Rajjoub Al-Mahdi, Álvaro Marco del Castillo, Javier Ramos Jiménez, Luis Borrego Bernabé, Fernando Arribas Ynsaurriaga, Rafael Salguero-Bodes

**Affiliations:** Cardiology Department, Hospital Universitario 12 de Octubre, Glorieta de Malaga 11, 28041 Madrid, Spain; Instituto de Investigación Hospital 12 de Octubre (imas12), Glorieta de Malaga 11, 28041 Madrid, Spain; Cardiology Department, Hospital Universitario 12 de Octubre, Glorieta de Malaga 11, 28041 Madrid, Spain; Instituto de Investigación Hospital 12 de Octubre (imas12), Glorieta de Malaga 11, 28041 Madrid, Spain; Cardiology Department, Hospital Universitario 12 de Octubre, Glorieta de Malaga 11, 28041 Madrid, Spain; Instituto de Investigación Hospital 12 de Octubre (imas12), Glorieta de Malaga 11, 28041 Madrid, Spain; Cardiology Department, Hospital Universitario 12 de Octubre, Glorieta de Malaga 11, 28041 Madrid, Spain; Instituto de Investigación Hospital 12 de Octubre (imas12), Glorieta de Malaga 11, 28041 Madrid, Spain; Cardiology Department, Hospital Universitario 12 de Octubre, Glorieta de Malaga 11, 28041 Madrid, Spain; Instituto de Investigación Hospital 12 de Octubre (imas12), Glorieta de Malaga 11, 28041 Madrid, Spain; Cardiology Department, Hospital Universitario 12 de Octubre, Glorieta de Malaga 11, 28041 Madrid, Spain; Instituto de Investigación Hospital 12 de Octubre (imas12), Glorieta de Malaga 11, 28041 Madrid, Spain; Cardiology Department, Hospital Universitario 12 de Octubre, Glorieta de Malaga 11, 28041 Madrid, Spain; Instituto de Investigación Hospital 12 de Octubre (imas12), Glorieta de Malaga 11, 28041 Madrid, Spain; Cardiology Department, Hospital Universitario 12 de Octubre, Glorieta de Malaga 11, 28041 Madrid, Spain; Instituto de Investigación Hospital 12 de Octubre (imas12), Glorieta de Malaga 11, 28041 Madrid, Spain; Department of Medicine, Universidad Complutense, Madrid, Spain; Centro de Investigación Biomédica en Red de enfermedades CardioVasculares (CIBERCV), Instituto de Salud Carlos III (ISCIII), Madrid, Spain; Cardiology Department, Hospital Universitario 12 de Octubre, Glorieta de Malaga 11, 28041 Madrid, Spain; Instituto de Investigación Hospital 12 de Octubre (imas12), Glorieta de Malaga 11, 28041 Madrid, Spain; Department of Medicine, Universidad Complutense, Madrid, Spain; Centro de Investigación Biomédica en Red de enfermedades CardioVasculares (CIBERCV), Instituto de Salud Carlos III (ISCIII), Madrid, Spain

**Keywords:** Atrial fibrillation, Pulsed field ablation, Pulmonary vein isolation, Durability, Systematic invasive remapping

## Abstract

**Aims:**

Data on long-term durability of pulmonary vein isolation (PVI) with newer pulsed field ablation (PFA) platforms and incremental value of electroanatomic mapping (EAM) guidance remain limited. This study evaluated chronic PVI durability following ablation with a balloon-in-basket PFA catheter and compared outcomes of fluoroscopy-only vs. fluoroscopy plus EAM workflows.

**Methods and results:**

In this prospective, single-centre trial, consecutive patients with paroxysmal or persistent atrial fibrillation (AF) underwent *de novo* PVI with a balloon-in-basket PFA catheter. Patients were randomized 1:1 to fluoroscopy-only or fluoroscopy plus EAM workflow. Systematic invasive remapping was planned >30 days after the index procedure. The dual primary efficacy endpoint was the proportion of pulmonary veins (PVs) and of patients with all PVs durably isolated. The prespecified secondary endpoint was the comparison of procedural characteristics and PVI durability between workflows. Safety outcomes included major adverse events within 30 days of index or remapping procedures. Ninety-six patients (67.4 ± 9.2 years, 44.8% female, 57.3% with paroxysmal AF) underwent index ablation. Fluoroscopy time and number of applications were 3.4 [2.4–4.6] minutes and 15 [13–16], respectively. Systematic remapping was performed in 86 patients at a median of 41 days, with per-vein and per-patient durability of 341/350 (97.4%), and 79/86 (91.9%) respectively. PVI durability and chronic remapping parameters were comparable between guidance strategies. No major adverse events occurred.

**Conclusion:**

A standardized balloon-in-basket PFA workflow for PVI achieved high PVI durability. EAM guidance did not confer additional procedural or durability benefit over fluoroscopy-only guidance.

## Graphical Abstract

**Graphical Abstract:**
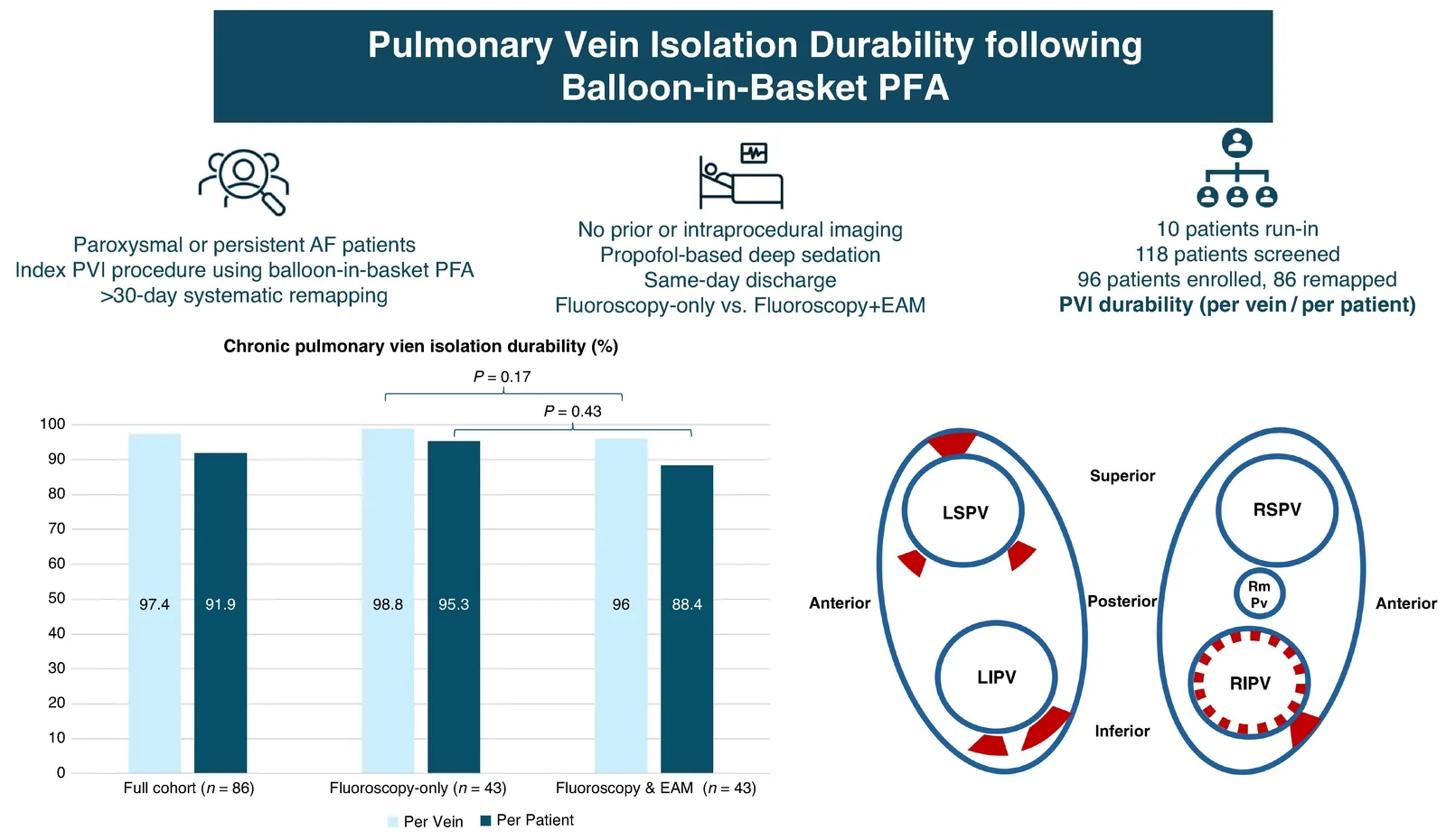

## Introduction

Atrial fibrillation (AF) is increasingly prevalent and has driven a progressive expansion in the use of catheter ablation, with pulmonary vein isolation (PVI) established as the cornerstone of rhythm control strategies.^1–3^

Pulsed field ablation (PFA) has rapidly gained adoption for AF ablation.^4,5^ First-in-human studies showed high success in achieving durable PVI.^6^ With broader implementation, long-term effectiveness has proven more variable, with poor tissue contact or uneven electrical field distribution as the main potential causes for the occurrence of reversible lesions, leading to outcomes comparable to those achieved with conventional thermal ablation technologies.^7–13^ In contrast, a consistent advantage of PFA has been its safety profile, particularly the reduction in collateral tissue injury inherent to its non-thermal ablation mechanism. At the same time, wider use has led to the recognition of specific procedure-related complications, including coronary vasospasm and haemolysis.^4,14^

Lesion formation and durability with PFA are determined by multiple procedural, waveform and device-related factors, including energy delivery, catheter design, and catheter–tissue interaction. In particular, electrode configuration and geometry influence the spatial distribution of the electric field.^15,16^ Accordingly, a wide range of catheter platforms has been developed with distinct designs aimed at optimizing pulmonary vein (PV) engagement and energy delivery.^5,17,18^

The balloon-in-basket PFA catheter represents a novel approach to PVI. The system can be fully integrated within a three-dimensional electroanatomic mapping (EAM) platform and incorporates impedance-based tissue proximity assessment, enabling real-time evaluation of catheter-tissue contact. Clinical performance of this technology has been evaluated, demonstrating high rates of freedom from atrial tachyarrhythmia recurrence with a favourable safety profile.^19,20^ However, data on lesion durability from systematic remapping, and specific guidance on workflow to optimize outcomes have not been reported.^8,21–23^

This study aimed to evaluate long-term durability after PVI following a specific workflow designed for the balloon-in-basket PFA catheter, while comparing outcomes of fluoroscopy-only vs. additional EAM-guidance strategies.

## Methods

### Study design

This prospective, single-centre trial evaluated the chronic durability of PVI using a balloon-in-basket PFA catheter with systematic remapping performed >30 days after the index procedure. As a secondary endpoint, patients were randomized to compare two guidance strategies. Operators were not blinded to treatment allocation; however, patients and endpoint adjudication were blinded. The study was conducted at a tertiary referral centre.

The protocol was approved by the institutional ethics committee (IRB approval 23/208.3) and complied with the Declaration of Helsinki. All participants provided written informed consent. The study was registered at ClinicalTrials.gov (identifier: NCT 07118046).

### Study population

After a run-in phase of 10 cases, consecutive patients with paroxysmal or persistent AF undergoing *de novo* catheter ablation were prospectively enrolled between August 2025 and February 2026.

Inclusion criteria were age ≥18 years, documented AF, and willingness and ability to provide written informed consent and comply with all study requirements. Key exclusion criteria included AF due to reversible or non-cardiac causes, prior left atrial (LA) ablation or surgery, anticipated need for ablation beyond the PVs, New York Heart Association class IV, or inability to complete follow-up. The full list of inclusion and exclusion criteria is provided in Supplementary material online, *Table S1*.

For the prespecified secondary endpoint, eligible patients were randomized 1:1 to PVI guided by fluoroscopy alone (control) or fluoroscopy plus EAM (experimental). Randomization was performed using a centralized, web-based system (REDCap) with a computer-generated permuted block sequence stratified by AF type (paroxysmal vs. persistent).

### Study endpoints

The study had a dual primary efficacy endpoint, comprising: (i) the proportion of PVs durably isolated; and (ii) the proportion of patients with all PVs durably isolated, both assessed >30 days after the index procedure.

Secondary endpoints included comparisons of procedural characteristics and PVI durability according to guidance strategy. A predefined exploratory secondary analysis assessed the association between durable PVI and impedance-based catheter–tissue proximity indicators recorded during the index procedure.

The primary safety endpoint was a composite of major adverse events, including cardiac perforation, cardiac tamponade, stroke or transient ischaemic attack, peripheral thromboembolic events, vascular complications requiring intervention, myocardial infarction, death occurring within 30 days of either the index or remapping procedures.

Primary efficacy and safety endpoints were adjudicated by an independent committee blinded to index procedure characteristics, who reviewed acute and chronic LA maps to determine PVI status and classify adverse events.

### Ablation system

The Volt™ PFA catheter and system have been previously described^19,20^; briefly, it comprises a 12.5-F over-the-wire balloon-in-basket PFA catheter (Volt™ PFA Catheter Sensor Enabled™, Abbott, Abbott Park, Illinois, USA), a 13-F steerable sheath (Agilis™ NxT Steerable Introducer Dual-Reach™), a dedicated PFA generator (Volt™ PFA Generator), and a compatible EAM platform (EnSite™ X EP System with PFA module, Abbott).

The balloon is inflated to a maximum diameter of 28 mm. with saline or a saline–contrast mixture to enable fluoroscopic visualization, and the catheter incorporates magnetic sensors for real-time positioning within the mapping system. The basket comprises eight evenly spaced insulated nitinol splines, with sensing and pacing capabilities, each with a 17.7-mm active electrode extending from the equatorial to the distal portion of the balloon. The system provides impedance-based catheter–tissue proximity information for each individual spline. This includes a colour-coded proximity display, which considers optimal contact when a change >16% from baseline impedance value is reached for each spline, and an active contact indicator that indicates impedance variations even below the 16% threshold. Pulsed electric fields are delivered in a bipolar, biphasic manner between sequential electrode pairs using R-wave–gated pulse trains. Standard ablation consists of applications of a 1.800 V waveform.

### Periprocedural management

Patients undergoing PVI with persistent AF, and those with paroxysmal AF and a CHA_2_DS_2_-VA score >1, underwent transoesophageal echocardiography within 72 h prior to the index ablation, but not the systematic remapping, procedure to exclude intracardiac thrombus. All procedures were performed under uninterrupted or minimally interrupted oral anticoagulation.

Vascular haemostasis was achieved using a figure-of-eight suture. Oral anticoagulation was resumed 4 h after ablation and continued for at least 2 months or according to individual thromboembolic risk. Patients previously on antiarrhythmic drug treatment discontinued them 2 months after index ablation. Patients were discharged 6 h after the ablation procedure in the absence of complications, or 4 h after remapping procedures.

### Index procedure systematic workflow

All procedures were performed under deep sedoanalgesia with propofol and fentanyl. Femoral venous access was obtained under ultrasound guidance. Unfractionated heparin was administered targeting an activated clotting time ≥300 s. A 1-mg bolus of atropine was given to prevent vagally mediated bradycardia, except in patients with a cardiac implantable electronic device with ventricular pacing capability. Transseptal puncture was performed under fluoroscopic guidance using an SL-0 sheath and a Brockenbrough needle, followed by exchange for a deflectable sheath (Agilis™ NxT, Abbott). The Volt™ PFA catheter was advanced through the sheath into the left superior PV over a J-tip guidewire. Baseline impedance values were obtained through catheter rotation in the LA, ensuring all splines were floating in the blood pool at some point during this process. Subsequently, the catheter was placed at the antrum of the left superior PV. For every PV, prior to PFA delivery, a position with optimal contact—i.e.: a >16% change in impedance from baseline coded by the system as a change in colour—in all splines was pursued. Spline placement was considered acceptable even below that 16% impedance-change threshold if contact indicators showed impedance variations synchronous with the patient’s heartbeat or respiration, a circumstance we termed SIV (Synchronous Impedance Variations). The presence of SIV was determined by the operator based on the information available on the contact-indicator display present both in the PFA generator and the EAM screen, which represent impedance variations as displacement of the contact-indicator bars moving between the inner and outer circles, representing zero and 16% impedance change from baseline, respectively. If the pace of the bars’ movement was stable, simultaneous and compatible with patient’s breathing or heartbeat, SIV was considered present. Absence of movement, as well as non-reproducible movements of the indicator bars, was not considered SIV. This definition, as well as integration into operator workflow, was developed during the 10-case run in phase.

A minimum of three applications were delivered in each vein with a fully inflated balloon. For the first two applications, a contralateral X-Ray projection (i.e.: right anterior oblique for left-sided PVs and left anterior oblique for right-sided PVS) was used to ensure sheath-catheter-guidewire alignment and to guide catheter rotation between the first and second application, aiming at positioning splines for the second application in the inter-spline spaces left in the initial one. A third application was systematically delivered towards the carina, that is, maintaining the guidewire inside the PV but deflecting the sheath to place the balloon in an inferior-posterior direction—for superior PVs—and superior-anterior direction—for inferior PVs. In the absence of SIV in any given spline, that is, if contact indicators were completely immobile, an additional application was delivered specifically targeting that region of the PV. In all cases left common PVs were treated as separate veins, and right intermediate veins were targeted independently. A summary of the main aspects of the workflow is provided in *Table 1*.

**Table 1:** Summary of key features of the implemented workflow for Balloon-in-Basket PFA pulmonary vein isolation

Aim	Action	Input
**Stable catheter-tissue contact**	Deflectable sheath directed to PV ostium Advancement of catheter alone (left-sided PVs) or catheter-sheath (right-sided PVs) towards PV ostium	Fluoroscopy (LAO 30° for left-sided, RAO 20° for right-sided PVs) Contact indicators on PFA generator and/or EAM-system
**Homogeneous pulsed field distribution**	Guidewire/catheter/sheath alignment on contralateral X-ray and/or EAM projection Catheter rotation for complementary spline positions between first and second application	Fluoroscopy (RAO 30° for left-sided, LAO 30° for right-sided PVs) ± 15–20°cranial tilt for superior PVs EAM (when present) to guide spline placement
**Reducing risk of gaps at regions with possible poor contact**	Identification of splines with absence of movement in impedance variation markers Additional application with sheath/catheter deflection towards regions with poor contact	Contact indicators on PFA generator and/or EAM-system EAM-guidance: spline identification on EAM Fluoroscopy-only guidance: sheath/catheter deflection to identify splines with poor contact
**Preventing carinal gaps and improving wide-antral lesion profile**	Systematic applications with sheath deflection and torque towards carina from both superior and inferior PVs	Fluoroscopy (RAO 30° for left-sided, LAO 30° for right-sided PVs) ± EAM

EAM, electroanatomic mapping; LAO, left anterior oblique; PFA, pulsed-field ablation; PV, pulmonary vein; RAO, right anterior oblique.

After the final PFA application, acute remapping was performed in all patients using the Volt catheter. Detected reconnections were targeted with additional PFA applications; these were classified as reconnections for the primary efficacy analysis.

#### Fluoroscopy-only guidance

The workflow included measures to aim for minimizing fluoroscopy use, previously developed and validated for cryoballoon ablation.^24^ As such, single-frame or short-duration fluoroscopy exposures were used to guide catheter manipulation and ablation steps according to procedural requirements (see Supplementary material online, *Figure S1*). Orthogonal fluoroscopic projections were systematically used for each vein. An ipsilateral projection (i.e.: left anterior oblique (LAO) 30° for left-sided PVs and right anterior oblique (RAO) 20° for right-sided PVs) was used initially to assess guidewire progression inside the PV and catheter approach to the antrum. Subsequently, a contralateral projection (i.e.: RAO 30° for left-sided PVs and LAO 30° for right-sided PVs) was used to assess sheath-catheter-guidewire alignment and spline distribution prior to applications, as well as to guide catheter positioning towards the carina. Additional 15 to 20°caudal tilt was used in selected superior PVs, per operators’ preference. A reference image of the initial catheter position in the contralateral projection was acquired and displayed on a secondary screen to guide subsequent spline positioning, ensuring coverage of inter-spline regions from the initial application (see Supplementary material online, *Figure S2*). A PFA application was repeated if catheter displacement was suspected. Given the absence of radiopaque markers allowing identification of each spline, if a contact indicator was still, response to sheath and catheter manoeuvres was evaluated to identify the region of the PV antrum with suspected poor spline-tissue contact and guide additional applications when required.

#### Fluoroscopy plus electroanatomic mapping guidance

Fluoroscopy was used as described above. In addition, EAM guidance was used to optimize catheter–tissue contact, assist sheath and catheter manipulation, and guide rotational positioning for complementary spline deployment relative to the initial application. No full LA map was created before PFA delivery. Identification of the position of each spline in the EAM system was also used to determine regions with suspected poor spline-tissue contact and guide additional applications.

### Invasive systematic remapping in the chronic phase

Repeat invasive remapping was systematically performed, irrespective of clinical recurrence, more than 30 days after the index procedure, when PFA lesions have shown to be mature and fully representative of its long-term characteristics.^25–27^ High-density bipolar voltage maps were acquired using an Advisor™ HD Grid X catheter (Abbott) under EAM guidance (Ensite X, Abbott) to assess PVI.

Durable PVI was defined as the presence of bidirectional block. Entrance block was defined as the absence of PV electrograms. Exit block was defined as the absence of atrial capture during pacing within the PV at an output of 8.0 V/2.0 ms. A diagnostic catheter was used to differentiate far-field signals originating from the LA appendage or the superior vena cava when necessary.

Conduction gaps identified at remapping were ablated in the same procedure with an irrigated contact force–sensing radiofrequency catheter (TactiCath™, Abbott).

### Clinical follow-up

No protocol-mandated follow-up was performed beyond remapping. Clinical events were assessed according to routine clinical care.

### Sample size

This was an exploratory, single-centre durability study with protocol-mandated invasive remapping. The planned sample size was 96 patients, randomized 1:1 to fluoroscopy-only vs. fluoroscopy plus EAM guidance, after an initial 10-case run-in phase. This sample size, within the range of similar systematic invasive remapping studies evaluating other PFA platforms, was considered sufficient to provide an estimate of chronic PVI durability with acceptable precision at both the pulmonary vein and patient levels. The comparison between guidance strategies was considered exploratory and was not powered for definitive superiority testing.

### Statistical analysis

Continuous variables are presented as mean ± SD or median (IQR), as appropriate, and categorical variables as counts and percentages. Comparisons between groups were performed using the Student’s *t*-test or Mann–Whitney *U* test for continuous variables and the chi-square or Fisher exact test for categorical variables, as appropriate. Associations between PVI status and catheter–tissue proximity were explored descriptively and with Fisher exact testing due to the low number of reconnection events. Safety outcomes are reported descriptively. All tests were 2-sided, and a *P* value <0.05 was considered statistically significant. Analyses were performed using Python (version 3.11; Python Software Foundation).

## Results

### Baseline characteristics

**Figure 1:**
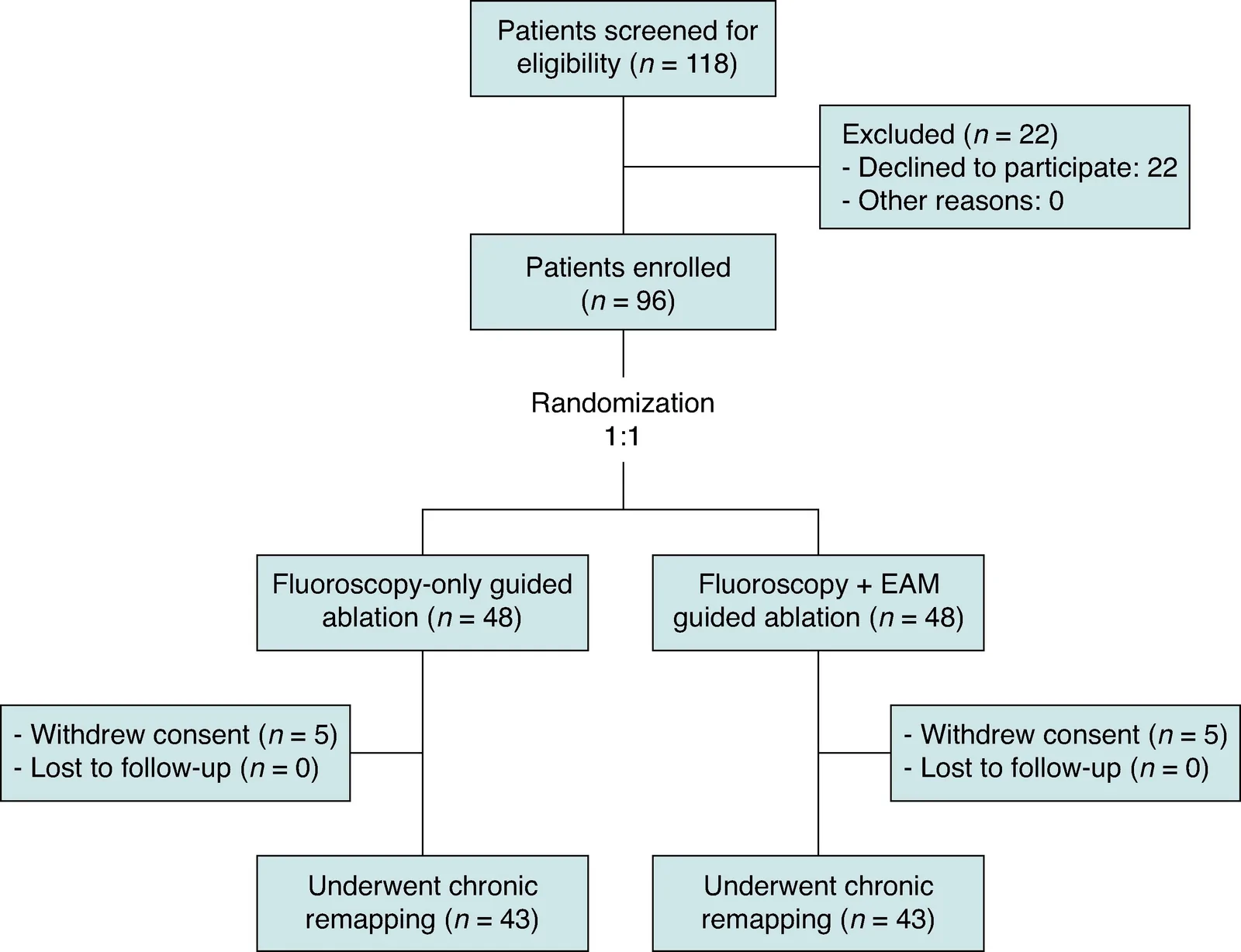
Study flow diagram. Patient flow from screening and enrolment to randomization (fluoroscopy-only vs. fluoroscopy plus electroanatomical mapping guidance) and systematic invasive remapping >30 days after the index procedure. EAM, electroanatomic mapping.

A total of 96 patients underwent index ablation, with 48 patients allocated to each guidance strategy. Of these, 86 underwent protocol-mandated chronic-phase remapping. The study flowchart is shown in *Figure 1*. The study population had a mean age of 67.4 ± 9.2 years, 43 patients (44.8%) were female, and 55 of them (57.3%) were diagnosed with paroxysmal AF. The most common comorbidities were hypertension in 61 patients (63.5%), diabetes mellitus in 21 (21.9%), heart failure in 20 (20.8%) and sleep apnoea in 14 (14.6%). The median CHA_2_DS_2_-VA score was 2 (IQR 1–3). Mean left ventricular ejection fraction was 59.1 ± 8.4%. Baseline characteristics for the overall cohort and by randomization group are shown in *Table 2*.

**Table 2:** Baseline characteristics according to randomization group

Variable	Overall cohort (*n* = 96)	Fluoroscopy− only (*n* = 48)	Fluoroscopy + EAM (*n* = 48)	*P*-value
Age, years	67.4 ± 9.2	68.5 ± 8.5	66.3 ± 9.8	0.245
Female sex, *n* (%)	43 (44.8)	23 (47.9)	20 (41.7)	0.53
BMI, kg/m^2^	30.0 ± 5.04	30.3 ± 4.7	29.7 ± 5.4	0.57
Paroxysmal AF, *n* (%)	55 (57.3)	28 (58.3)	27 (56.3)	1.00
Time from AF diagnosis to ablation, *n* (%)				
≤6 months	28 (29.2)	13 (27.1)	15 (31.3)	0.93
6–12 months	39 (40.6)	20 (41.7)	19 (39.6)	
>12 months	29 (30.2)	15 (31.3)	14 (29.2)	
EHRA score, *n* (%)				
EHRA I	13 (14,6)	5 (11.4)	8 (17.8)	
EHRA II	58 (65.2)	29 (65.9)	29 (64.4)	0.70
EHRA III–IV	18 (20,3)	10 (22.7)	8 (17.8)	
Not reported	7 (7.3)	4 (8.3)	3 (6.2)	
Hypertension, *n* (%)	61 (63.5)	31 (64.6)	30 (62.5)	1.00
Diabetes mellitus, *n* (%)	21 (21.9)	10 (20.8)	11 (22.9)	1.00
Sleep apnoea, *n* (%)	14 (14.6)	7 (14.6)	7 (14.6)	1.00
COPD (moderate–severe), *n* (%)	4 (4.2)	4 (8.3)	0 (0)	0.12
Prior stroke, *n* (%)	0 (0.0)	1 (2.3)	0 (0)	0.49
CHA_2_DS_2_-VA score, median (IQR)	2 (1–3)	2.0 (1.25)	2.0 (2.0)	0.34
LVEF, %	59.1 ± 8.4	58.5 ± 8.5	59.7 ± 8.3	0.46
Left atrial dilation, *n* (%)				
No	49 (51.0)	26 (54.2)	23 (47.9)	
Mild	24 (25.0)	10 (20.8)	14 (29.2)	0.73
Moderate	13 (13.5)	6 (12.5)	7 (14.6)	
Severe	10 (10.4)	6 (12.5)	4 (8.3)	
Heart failure, *n* (%)	20 (20.8)	11 (22.9)	9 (18.8)	0.80
Prior cardiac surgery, *n* (%)	2 (2.1)	2 (4.2)	0 (0)	0.49
Ischaemic heart disease, *n* (%)	10 (10.4)	5 (10.4)	5 (10.4)	1.00
Cardiomyopathy, *n* (%)	15 (15.6)	7 (14.6)	8 (16.7)	1.00
Antiarrhythmic drug use, *n* (%)	18 (18.75)	10 (20.8)	8 (16.7)	0.79

Values are presented as mean ± SD, median (IQR), or *n* (%).

AF, atrial fibrillation; BMI, body mass index; COPD, chronic obstructive pulmonary disease; EHRA, European Heart Rhythm Association; EAM, electroanatomical mapping; IQR, interquartile range; LA, left atrium; LVEF, left ventricular ejection fraction.

### Index procedure characteristics

Mean procedure time was 63.0 ± 15.9 min and mean LA dwell time was 37.6 ± 10.3 min. Median fluoroscopy time was 3.4 min (IQR 2.4–4.6), dose–area product was 3.19 Gy·cm^2^ (IQR 1.69–5.57), and the median number of applications was 15 (IQR 13–16).

Acute remapping was performed in 88 patients and confirmed acute PVI of 100%. Median acute remapping time was 12.5 min (IQR 11.0–15.0), with a mean of 2020 ± 1333 electrogram points acquired (*Table 3*).

**Table 3:** Index procedure characteristics

	Index procedure			
	Overall cohort (*n* = 96)	Fluoroscopy− only (*n* = 48)	Fluoroscopy+ EAM (*n* = 48)	*P*-value
Procedure time, min	63.0 ± 15.9	62.9 ± 16.3	63.1 ± 15.7	0.96
LA dwell time, min	37.6 ± 10.3	37.6 ± 11.0	37.6 ± 9.8	0.99
Fluoroscopy time, min	3.4 [2.4–4.6]	3.6 [2.5–4.5]	3.2 [2.3–4.6]	0.78
Dose–area product, Gy·cm^2^	3.2 [1.7–5.6]	3.04 [1.6–5.8]	3.63 [1.8–5.2]	0.69
Number of applications	15 [13–16]	14 [13–16.2]	15 [14–15.2]	0.89
	**Acute electroanatomic mapping (balloon-in-basket catheter)**			
Acute remapping time, min	12.5 [11–15]	12 [11–16]	13 [11–14]	0.32
Number of electrogram points acquired	1337 [1181–1585]	1321 [1180–1585]	1346 [1229–1586]	0.53

Values are presented as mean ± SD or median [IQR], as appropriate.

EAM, electroanatomical mapping; IQR, interquartile range; LA, left atrium; SD, standard deviation.

### Primary efficacy endpoint

**Figure 2:**
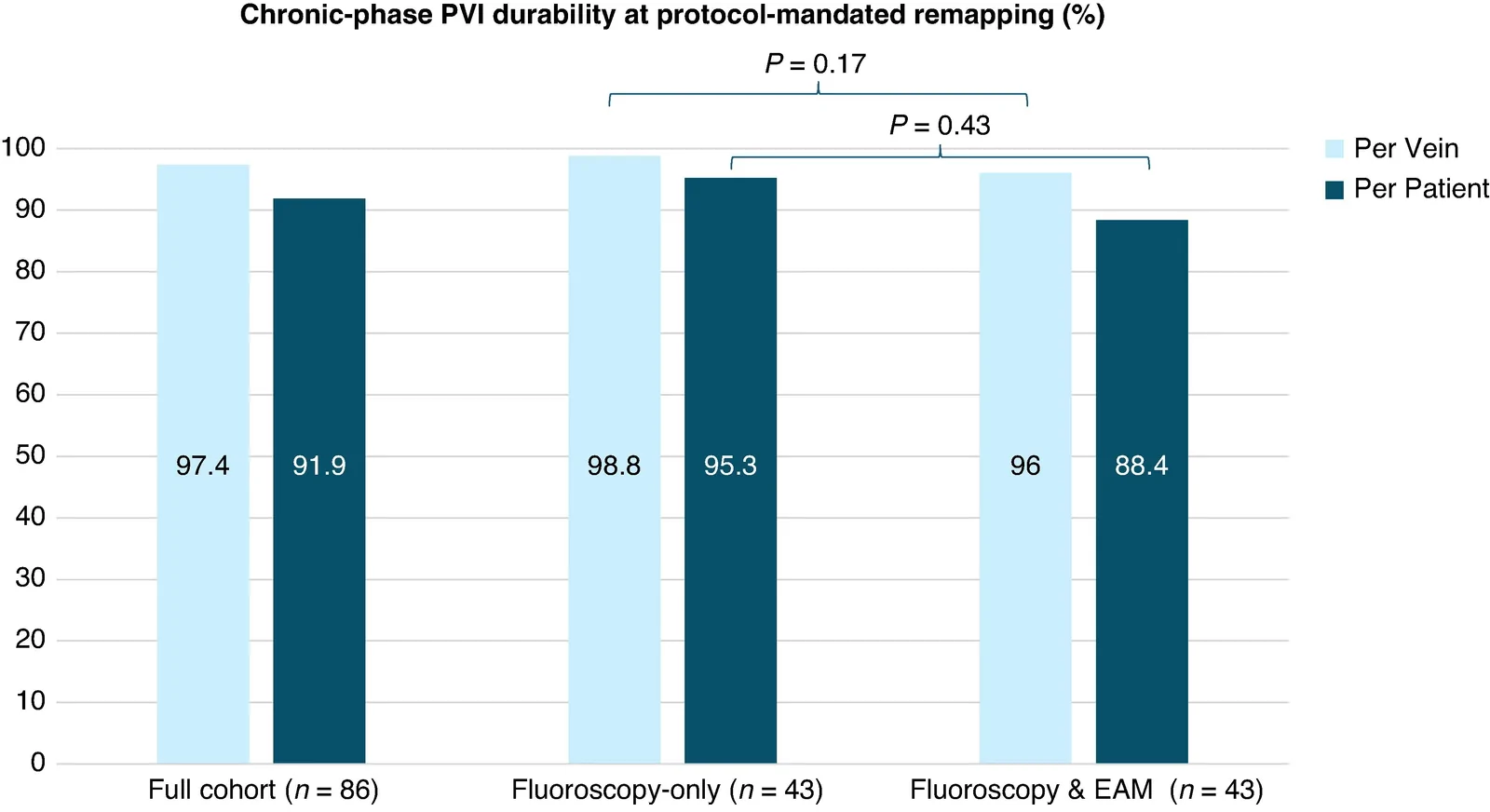
Chronic-phase PVI durability at protocol-mandated remapping. Pulmonary vein-level and patient-level PVI durability are shown for the overall remapping cohort and stratified by procedural guidance strategy. No significant differences were observed between the fluoroscopy-only and fluoroscopy plus EAM groups at the PV level (98.8% vs. 96.0%; *P* = 0.17) or patient level (95.3% vs. 88.4%; *P* = 0.43). EAM, electroanatomic mapping; PV, pulmonary vein; PVI, pulmonary vein isolation.

Invasive remapping was performed in 86 patients a median of 41 (IQR 34–54) days after the index procedure. The mean number of electrogram points acquired was 4166 ± 1152. Median LA dwell time for remapping time was 16 min (IQR 14–19), and median fluoroscopy time during remapping was 1.3 min (IQR 0.9–2.5) (*Table 3*). Durable PVI was observed in 341 of 350 (97.4%) PVs, and 79 of 86 (91.9%) patients had all PVs isolated (*Figure 2*).

**Figure 3:**
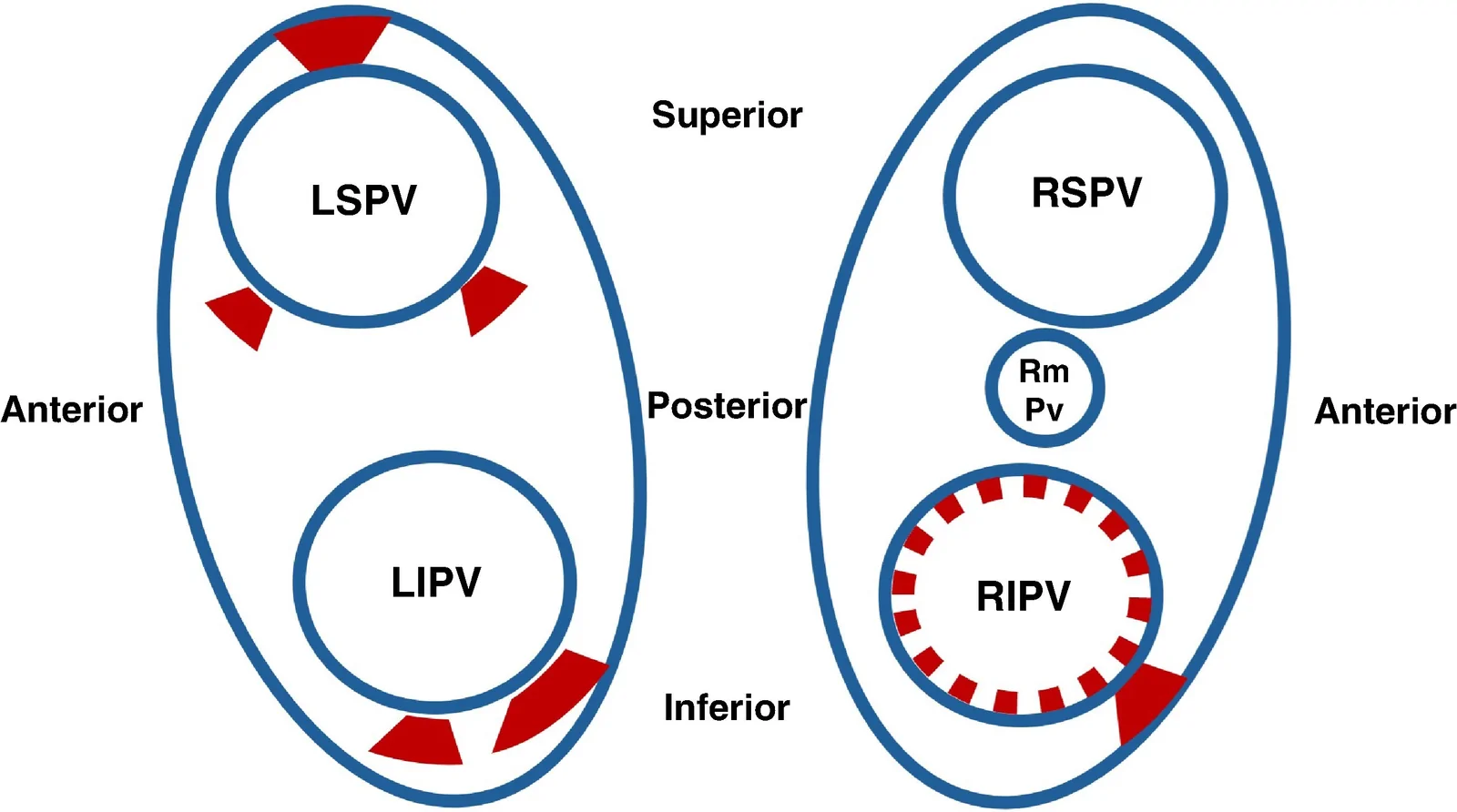
Gap location among patients with PV reconnection. Schematic representation of gap location in a modified postero-anterior view of the left atrium. Dotted line in the right inferior pulmonary vein indicates presence of preserved electrograms around all the pulmonary vein ostium. Both gaps in the inferior part of the left inferior pulmonary vein led to reconnection of both left-sided veins. LIPV, left inferior pulmonary vein; LSPV, left superior pulmonary vein; RIPV, right inferior pulmonary vein; RmPV, right intermediate pulmonary vein; RSPV, right superior pulmonary vein.

Reconnection was observed at the left inferior PV in 3 cases, the left superior PV in 2 cases, and the right inferior PV in 2 cases. In two patients, a single gap led to reconnection of both the superior and inferior left-sided PVs. No reconnections were observed in the right superior PV (*Figure 3*). A left common PV was present in 6 patients, all of whom showed durable isolation.

At the time of remapping, atrial arrhythmia recurrence was observed in 6 patients (7.0%), including atrial tachycardia in 1 patient, left atrial flutter in 2 patients (both, roof-dependent) and AF in 3 patients. No PV reconnections were observed among these patients.

### Secondary endpoints

At chronic-phase remapping, PVI durability was observed in 171 of 173 (98.8%) and 170 of 177 (96.0%) PVs in the fluoroscopy-only and fluoroscopy plus EAM groups, respectively (*P* = 0.17). Durable isolation of all PVs was observed in 41 of 43 (95.3%) and 38 of 43 (88.4%) patients in the fluoroscopy-only and fluoroscopy plus EAM groups, respectively (*P* = 0.43).

Index procedure characteristics were also similar between groups, with no significant differences in procedure time (62.9 ± 16.3 vs. 63.1 ± 15.7 min; *P* = 0.96), LA dwell time (37.6 ± 11.0 vs. 37.6 ± 9.8 min; *P* = 0.99), fluoroscopy time [3.6 (IQR: 2.5–4.5) vs. 3.2 (IQR: 2.3–4.6) minutes; *P* = 0.78], dose–area product [3.04 (IQR: 1.6–5.8) vs. 3.63 (IQR: 1.8–5.2) Gy·cm^2^; *P* = 0.69], or number of applications [14 (IQR: 13–16.2) vs. 15 (IQR: 14–15.2); *P* = 0.89] (*Table 3*).

Acute remapping time [12 (IQR: 11–16) vs. 13 (IQR: 11–14) minutes; *P* = 0.32] and the number of electrogram points acquired [1321 (IQR: 1180–1585) vs. 1346 (IQR: 1229–1586); *P* = 0.53] were also similar between groups. Likewise, at chronic-phase remapping, the interval from index ablation to remapping [45 (IQR: 34–55) vs. 41 (IQR: 34–53) days; *P* = 0.48], remapping time [16 (IQR: 14–19) vs. 16 (IQR: 14–19) minutes; *P* = 1.00], number of electrogram points acquired (4142 ± 1156 vs. 4189 ± 1163 points; *P* = 0.86), and fluoroscopy time [1.33 (1–2.60) vs. 1.15 (0.8–2.4) minutes; *P* = 0.22] were similar between groups (*Table 4*).

**Table 4:** Characteristics of chronic remapping procedure

	Chronic-phase remapping procedure			
	Overall cohort (*n* = 86)	Fluoroscopy− only (*n* = 43)	Fluoroscopy + EAM (*n* = 43)	*P*-value
Days from index ablation	41 [34–54]	45 [34–55]	41 [34–53]	0.48
LA dwell time, min	16 [14–19]	16 [14–19]	16 [14–19]	1.00
Fluoroscopy time, min	1.3 [0.9–2.5]	1.3 [1–2.60]	1.1 [0.8–2.4]	0.22
Number of electrogram points acquired	4166 ± 1152	4142 ± 1156	4189 ± 1163	0.86

Values are presented as mean ± SD or median [IQR], as appropriate.

EAM, electroanatomical mapping; IQR, interquartile range; LA, left atrium; SD, standard deviation.

At the time of remapping, atrial tachyarrhythmia recurrence was observed in 3 patients (7.0%) in each arm.

### Distribution of gaps and catheter–tissue proximity analysis

Among PV segments with SIV despite an impedance change below the 16% threshold, 348 of 349 (99.7%) showed no reconnection, a rate similar to that observed in segments with optimal contact (2464 of 2468, 99.8%; *P* = 0.48). In one reconnected PV, contact feedback revealed no impedance variations (i.e.: motionless contact indicator) in the segment where the gap was identified, and another reconnected PV showed a large ostial diameter (28 mm), with presence of electrograms at the ostium attributed to application having been delivered too distal inside the vein. A schematic representation of gap location is provided in *Figure 3*.

### Safety endpoint

No major adverse events occurred within 30 days of either the index or remapping procedures. One minor vascular access-site bleeding occurred after the index procedure and did not require intervention or prolonged hospitalization. A transient ST-segment elevation occurred during one remapping procedure and resolved without clinical sequelae.

### Run-in phase

Out of the first 10 patients treated with the balloon-in-basket catheter at the institution, between June and July 2025, 7 accepted to undergo systematic remapping. Only 1 reconnected PV was identified in a patient without clinical recurrence. One patient showed a completely preserved right-sided carina, leading to the implementation of dedicated applications in the workflow (see Supplementary material online, *Figure S3*).

## Discussion

In this study, we provide chronic-phase invasive durability data after PVI following a specific workflow designed for the balloon-in-basket PFA catheter. Outcomes of this systematic approach are similar when guided by fluoroscopy-only or with the addition of EAM guidance strategy.

### PVI durability

This is the first study to systematically evaluate PVI durability after PVI with the balloon-in-basket PFA catheter and to compare fluoroscopy-only vs. fluoroscopy plus EAM guidance in a randomized design. Previous data regarding outcomes with this catheter were limited to acute procedural success in both the feasibility cohort^19^ and the subsequent VOLT CE Mark study,^20^ both with >99% of PVs acutely isolated, and 12-month single-procedure freedom from documented atrial arrhythmia of 80.5% in paroxysmal and 63.3% in persistent AF patients.^28^ Owing to the nature of PFA, acute PVI is a poor indicator of chronic outcomes due to potential reversible electroporation or myocardial stunning rather than irreversible tissue injury, making chronic invasive remapping important to establish true lesion durability.^17,29^ In this regard, other preclinical PFA studies have assessed chronic lesion formation at ∼28–30 days,^25,26^ and prior clinical PFA remapping studies have also used remapping beyond 30 days to evaluate chronic-phase PVI durability.^30^ However, in the previous balloon-in-basket experience, PVI durability data were limited so far to 19 patients with clinically indicated repeat procedures, in whom 78.5% of PVs remained isolated, leading to 57.9% patients with all PVs isolated.^28^ These durability values could be misleading given the presumed negative selection bias of a population with atrial tachyarrhythmia recurrence and the relatively small sample size. In comparison, among the 6 patients with clinical recurrence in our cohort, we found 100% PVI durability at protocol-mandated remapping, which also may not reflect overall durability. This disparity between systematic vs. clinically indicated remapping has been widely reported with other single-shot PFA platforms, particularly with the pentaspline PFA catheter.^8,9,31^ In this context, the 97.4% PV-level durability and 91.9% patient-level complete PVI observed after *de novo* balloon-in-basket PFA in our larger cohort appear consistent with the upper range of durability reported in early PFA remapping studies. Besides, based on prior PFA cohorts in which high chronic PVI durability was accompanied by favourable 1-year outcomes,^8^ our findings provide a mechanistic basis to expect meaningful clinical benefit. The present study was not designed to assess clinical efficacy, and no protocol-driven long-term rhythm follow-up was performed. Consequently, the relationship between PVI durability and arrhythmia recurrence should be considered hypothesis-generating and warrants confirmation in dedicated longitudinal studies.

### From catheter design to procedural workflow

Durable PVI after single-shot PFA is influenced by multiple procedural and device-specific factors, including waveform design, electrode configuration, catheter geometry, catheter–tissue contact, and spatial overlap between consecutive applications. Together, these factors determine the homogeneity of antral energy delivery and the likelihood of avoiding residual gaps.^4,16^ Computer-modelling data have suggested that balloon-based PFA could offer more efficient target-focused energy delivery compared with other catheter designs, although clinical implications remain speculative.^32^ However, as outcomes cannot be generalized across different PFA platforms, also different workflows within the same platform can lead to markedly different results. A recent study comparing PVI durability between the balloon-in-basket and pentaspline catheters in a small series reported marked differences across platforms.^33^ In parallel, we have previously reported significant differences in outcomes achieved with the pentaspline catheter, within the same centre and operators, following workflow refinement.^30^

In our cohort, procedural workflow was tailored to catheter characteristics and refined before randomization through a 10-patient run-in phase, 7 of whom underwent chronic remapping. This early experience, together with substantial prior experience with balloon-based cryoablation and other PFA single-shot systems, may have helped achieve the high efficacy rates observed in this cohort.

Some of the key features of this workflow include: the choice of 3 applications per PV, with rotation between the first 2 applications to cover inter-spline regions, and a systematic carina-directed application; the delivery of additional treatment towards regions without sufficient contact information; variant anatomy addressed by treating left common PVs as separate veins and targeting right intermediate veins independently; orthogonal projections to ensure alignment and spline distribution; use of reference images to control catheter rotation; and repeat applications when displacement during the application was suspected. This structured workflow may help explain the durable isolation observed across PV anatomies and the comparable outcomes between fluoroscopy-only and EAM-guided procedures.

### Real-time continuous contact assessment

Adequate contact remains a key determinant of PFA lesion formation. Imaging and mapping tools such as ICE and EAM can improve visualization of catheter position and tissue engagement, although they add procedural complexity, cost, and may not be universally available. In our cohort, both the ≥16% impedance-change threshold considered ‘optimal contact’ and the presence of dynamic changes, termed by our team ‘synchronous impedance variations, or SIV’ due to the characteristic changes with heartbeat or respiration, showed similarly high rates of lesion durability. Our study provides the first systematic evaluation of these markers and may consequently help operators using this system to take better informed decisions on lesion delivery.

Although the study was not powered to demonstrate equivalence or non-inferiority between guidance strategies, the randomized comparison between fluoroscopy-only and fluoroscopy plus EAM guidance showed no statistically significant differences in procedural or chronic-phase durability outcomes. Reconnection events were numerically more frequent in the EAM-guided group; however the overall number of gaps was low, limiting the statistical power to detect between-group differences. Procedure time, fluoroscopy time, LA dwell time, and safety outcomes were also not significantly different between groups. These findings suggest that, within this standardized workflow, the use of EAM as an adjunctive navigational tool, without systematic creation of a full LA map to guide therapy delivery, was not associated with a measurable incremental benefit for PVI-only procedures with this catheter. This exploratory comparison should therefore not be interpreted as evidence of equivalence between strategies. Whether systematic creation of complete LA maps could facilitate more tailored lesion delivery in selected patients, particularly those with larger atria or PV ostia, remains to be evaluated in future studies.

Finally, safety outcomes were favourable, with no major complications during either index or remapping procedures. This aligns with prior data on the balloon-in-basket platform and supports the procedural safety of both the index and systematic remapping strategy in the present cohort.

### Limitations

This study has limitations. First, it was conducted at a single high-volume centre by operators with substantial prior experience in balloon-based and other PFA single-shot ablation systems, which may not reflect routine real-world implementation. Although the proposed workflow may be easily adoptable by other operators, variability in outcomes throughout its implementation cannot be ruled out and has been reported with other systems.^9,31^ Second, although systematic remapping was performed in most patients, the low number of reconnection events limited the power to compare guidance strategies, analyse vein-specific predictors, or validate impedance-based proximity markers. Accordingly, the absence of a significant difference between fluoroscopy-only and EAM-guided strategies should be interpreted cautiously and does not exclude a potential benefit of EAM in selected patients, anatomies, or less standardized workflows. Third, long-term clinical follow-up is beyond the scope of this analysis, which was centred on PVI durability outcomes, limiting assessment of the relationship between PVI durability and clinical recurrence for this specific cohort. Fourth, despite blinded endpoint adjudication as implemented, operators were not blinded to guidance strategy, which may have influenced procedural behaviour. Finally, dedicated assessments for subclinical safety events (i.e. systematic phrenic nerve pacing, post-procedural cerebral imaging, oesophageal evaluation, PV imaging, or laboratory testing for haemolysis), were not performed; however, these endpoints have been specifically evaluated in prior studies.^19,20^

## Conclusion

In this prospective single-centre study, the use of a structured workflow with the balloon-in-basket PFA platform was associated with high chronic-phase PVI durability at protocol-mandated remapping. No statistically significant differences in procedural or durability outcomes were observed between fluoroscopy-only and fluoroscopy plus EAM guidance. Impedance-based tissue-contact indicators were associated with chronic-phase PVI durability.

## Supplementary Material

euag244_Supplementary_Data

## Data Availability

The data underlying this article are available in the article. Further data will be shared upon reasonable request to the corresponding author.
