# Supplementary material for: Pulmonary vein isolation durability in a randomized trial comparing fluoroscopy-only vs. electroanatomic mapping strategies for pulmonary vein isolation with balloon-in-basket pulsed-field ablation
Source: Europace. 2026 Sep 3;28(9):euag244. doi: 10.1093/europace/euag244 (PMC13585463; doi:10.1093/europace/euag244)
Supplement: Supplementary file 1 [file euag244_supplementary_data.zip › Supplementary Table 1 .docx]

**Supplementary material**

**Supplementary Table 1. Inclusion and exclusion criteria.**

**Inclusion criteria**

Age ≥18 years

Documented atrial fibrillation (ECG or rhythm monitoring)

Clinical indication to undergo pulmonary vein isolation

**Exclusion criteria**

Atrial fibrillation secondary to reversible or noncardiac causes

Prior pulmonary vein isolation or left atrial ablation

Need for ablation beyond pulmonary vein isolation or additional electrophysiological study

Contraindication or intolerance to heparin

Presence of left atrial thrombus

New York Heart Association class III–IV heart failure

Recent major cardiovascular events (myocardial infarction, cardiac surgery or stroke within 90 days)

Congenital heart disease

Severe frailty or life expectancy <1 year

Pregnancy (ongoing or planned within 6 months)

Unwillingness or inability to provide informed consent

Participation in another interventional study interfering with outcomes
